# Astaxanthin Pretreatment Attenuates Hepatic Ischemia Reperfusion-Induced Apoptosis and Autophagy via the ROS/MAPK Pathway in Mice

**DOI:** 10.3390/md13063368

**Published:** 2015-05-27

**Authors:** Jingjing Li, Fan Wang, Yujing Xia, Weiqi Dai, Kan Chen, Sainan Li, Tong Liu, Yuanyuan Zheng, Jianrong Wang, Wenxia Lu, Yuqing Zhou, Qin Yin, Jie Lu, Yingqun Zhou, Chuanyong Guo

**Affiliations:** 1 Department of Gastroenterology, Shanghai Tenth People’s Hospital, Tongji University School of Medicine, Shanghai 200072, China; E-Mails: sealjj@126.com (J.L.); fairywong04285@163.com (F.W.); gagaxyj@126.com (Y.X.); dai_yue@163.com (W.D.); cutking@126.com (K.C.); Lrk678@126.com (S.L.); klmn1334@sina.com (T.L.); sxzhengyuanyuan@126.com (Y.Z.); kennisren@hotmai.com (J.L.); 2 The First Clinical Medical College of Nanjing Medical University, Nanjing 210029, China; E-Mails: hellowangjr@163.com (J.W.); 15214327248@163.com (W.L.); 3 The First Affiliated Hospital of Soochow University, Suzhou 215006, China; E-Mails: zyq937065339@163.com (Y.Z.); yinqin201011@163.com (Q.Y.)

**Keywords:** hepatic ischemia reperfusion, oxidative stress, astaxanthin, reactive oxygen species

## Abstract

Background: Hepatic ischemia reperfusion (IR) is an important issue in complex liver resection and liver transplantation. The aim of the present study was to determine the protective effect of astaxanthin (ASX), an antioxidant, on hepatic IR injury via the reactive oxygen species/mitogen-activated protein kinase (ROS/MAPK) pathway. Methods: Mice were randomized into a sham, IR, ASX or IR + ASX group. The mice received ASX at different doses (30 mg/kg or 60 mg/kg) for 14 days. Serum and tissue samples at 2 h, 8 h and 24 h after abdominal surgery were collected to assess alanine aminotransferase (ALT), aspartate aminotransferase (AST), inflammation factors, ROS, and key proteins in the MAPK family. Results: ASX reduced the release of ROS and cytokines leading to inhibition of apoptosis and autophagy via down-regulation of the activated phosphorylation of related proteins in the MAPK family, such as P38 MAPK, JNK and ERK in this model of hepatic IR injury. Conclusion: Apoptosis and autophagy caused by hepatic IR injury were inhibited by ASX following a reduction in the release of ROS and inflammatory cytokines, and the relationship between the two may be associated with the inactivation of the MAPK family.

## 1. Introduction

Hepatic ischemia reperfusion (IR) injury generally occurs in hemorrhagic shock, liver transplantation and other medical conditions and is a pathophysiological process influencing liver function after hepatic resection and severe trauma [1,2]. Whether hepatic IR injury can be effectively avoided is important in limiting progress in hepatobiliary surgery. Though the concept of IR was first proposed in 1960 by Jennings [3], there are still no effective prevention methods due to its complicated mechanism, although studies have shown that its development is related to liver Kupffer cells, reactive oxygen species (ROS), calcium overload, and inflammatory cytokines [4,5,6]. Research demonstrated that Toll-like receptor (TLR) stimulation by IR may play an important role in the development of new therapeutic strategies in liver Kupffer cells [7,8,9]. Therefore, the therapy of hepatic IR has become a focus of attention in the medical community.

Ischemia reperfusion is a multifactorial process and includes major oxidative stress induced by ischemia and hypoxia [10]. Under normal physiological conditions, the production and elimination of oxygen free bases is dynamically balanced. However, endothelial cells and Kupffer cells activated by oxidative stress can generate large numbers of ROS through nicotinamide adenine dinucleotide phosphate (NADPH) in the membrane, and ROS damage liver cells by changing the permeability of the cell membrane, causing lipid peroxidation or directly increasing neutrophil microcirculation [10,11,12]. In addition, cytokines such as TNF-α and IL-6, released by activated Kupffer cells and aggregated neutrophils, also play a key role in IR injury [13]. TNF-α promotes swelling of endothelial cells to activate ROS, while IL-6 can induce hepatocyte injury to produce C-reactive protein, α-trypsin and fibrinogen which are associated with the MAPK family, and the PI3K/Akt and HMGB1 pathways [14,15,16]. Kohli and colleagues found that the death of liver sinusoidal endothelial cells and hepatocytes was by apoptosis, and intrinsic apoptosis induced by the explosion of ROS and inflammatory cytokines resulted in a mitochondrial energy metabolism disorder which may lead to hepatic IR injury [2,17]. However, the ratio of pro-apoptotic Bax and anti-apoptotic Bcl-2, located in the outer membrane, in mitochondrial apoptosis determines cell survival and death by controlling the opening and closing of the mitochondrial permeability transition pore (MPTP), as described by Selzner, Imahashi and colleagues [18,19].

Autophagy, another type of programmed cell death, has significant differences in biochemical pathways and morphology compared with apoptosis. Recent studies have found that autophagy can be regulated by a molecular mechanism and coordination with apoptosis to promote cell death [20]. Forty years ago, Sybers and colleagues first observed an increase in autophagic vacuoles following IR [21]. In addition, autophagy induced by cardiac ischemia and strengthened by reperfusion injury was found in rabbits by Decker *et al.* [22]. These studies provided a strong basis for the role of autophagy in hepatic IR. In 1998, Liang *et al.* demonstrated that elimination of the autophagy gene, Beclin-1, could inhibit the replication of the Sindbis virus when it was combined with Bcl-2, the decline of which promoted autophagy [23]. Therefore, the mechanism of hepatic IR injury may be further elucidated by evaluating the interaction between autophagy and apoptosis.

As autophagy and apoptosis are induced by the release of ROS, this is important in the IR injury process, and scavenging ROS may be a target when screening drugs. Astaxanthin (3,3′-dihydroxy-β, β′-carotene-4,4′-dione, ASX), a carotenoid which is found in fresh-water microalgae and marine organisms including phytoplankton and fish, has higher antioxidant activity than lutein, α-carotene and β-carotene [24]. The anti-lipid oxidation, anti-inflammation, anti-diabetes and anti-cancer effects of ASX have aroused increased attention both nationally and internationally [25,26,27]. Research has shown that ASX can exert strong antioxidant activity by quenching singlet oxygen and purifying free radicals in ulcer and hepatic stellate cells [28,29]. In alveolar epithelial cells, ASX blocked ROS generation and dose-dependently inhibited apoptosis through a mitochondrial signaling pathway, as shown by Song and colleagues [30]. ASX treatment has been shown to have therapeutic properties, as U937 cells were protected from oxidative stress caused by lipopolysaccharide thus reducing ROS production [31]. In other system diseases, tissues and cells, ASX also effectively resisted oxidative stress via the inhibition of ROS release caused by UVB in human keratinocytes, neutrophils treated with free fatty acids and high glucose, and by alloxan in diabetic rats [32,33,34]. With respect to IR, Lu, Shen and Lauver showed the protective effects of ASX on brain and myocardial injury following IR [35,36,37]. However, in hepatocellular injury following IR, Gulten *et al.* found that ASX treatment significantly decreased the conversion of xanthine dehydrogenase (XDH) to xanthine oxidase (XO), which reduced the level of oxidative stress [38]. In another study, Ping *et al.* illustrated that cryptotanshinone alleviated liver IR injury via anti-mitochondrial apoptosis [39]. It is unknown whether ASX can protect hepatocytes by directly reducing ROS and the pathways mediating the interaction between ROS, apoptosis and autophagy.

The aim of the present study was to determine the protective mechanism of ASX on hepatic IR injury. It is hypothesized that ASX pretreatment can attenuate ROS production induced by IR to down-regulate apoptosis and autophagy and achieve liver function protection and rapid injury recovery.

## 2. Results and Discussion

### 2.1. ASX Had No Effect on Normal Liver Tissue

**Figure 1:**
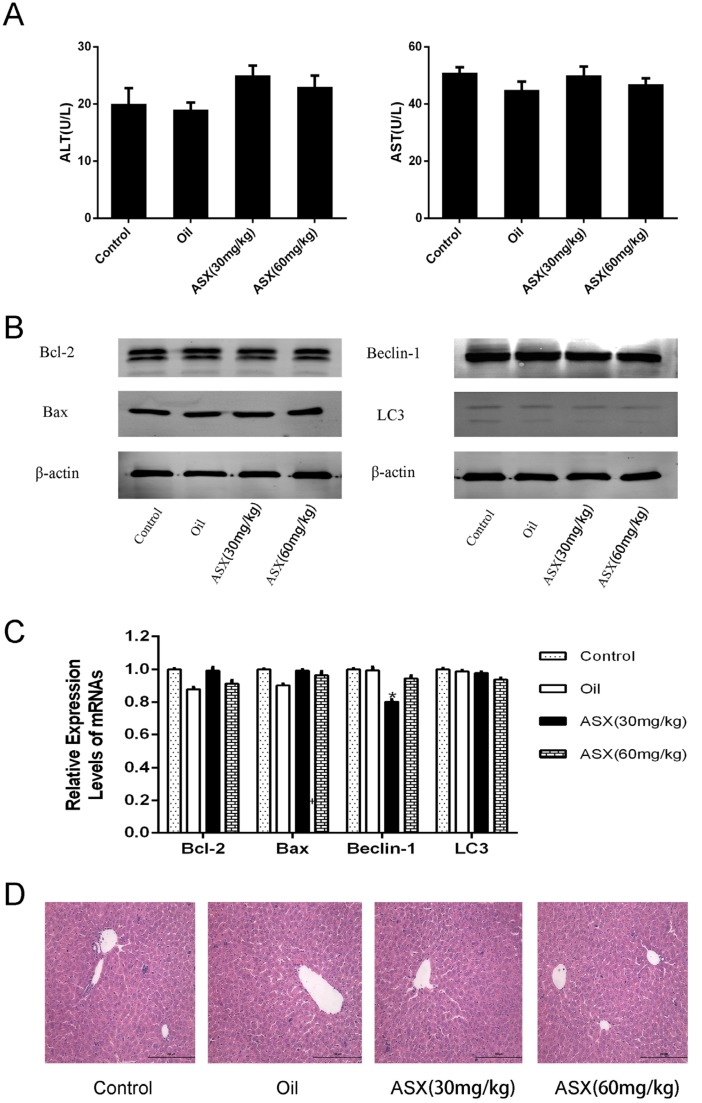
ASX had no injurious effects on liver tissue. (**A**) ALT and AST were expressed as the mean ± SD of 8 mice per group; (**B**) western blots of the expression of Bcl-2, Bax, Beclin-1 and LC3 are shown by gray bands; (**C**) the mRNA expression of Bcl-2, Bax, Beclin-1 and LC3 was assessed by real time PCR. The experiments were repeated three times and data are presented as the mean ± SD. * *p* < 0.05 for ASX (30 mg/kg) *vs.* oil; (**D**) representative hematoxylin and eosin (HE) stained sections of liver shown by digital microscopy. Original magnifications: 200×.

Before validation of the protective effects of ASX on hepatic IR injury, we first determined the influence of ASX on normal liver tissue. The same number of mice were given the same volume of saline, olive oil or ASX (30 mg/kg or 60 mg/kg) for 14 days, respectively. Serum and liver tissues were obtained from the mice to examine liver function, pathology and markers related to damage (Bcl-2, Bax, Beclin-1 and LC3) using biochemical methods, PCR detection and western blot. The results showed that the expression of serum liver enzymes in the four groups was close to normal levels (Figure 1A), while at the gene and protein levels, differences in relevant autophagic and apoptotic indicators were not obvious (Figure 1B,C). Finally, we identified pathological and morphological changes in the ASX groups. The cell structures showed small disturbances, which may have been due to the influence of drug metabolism, and liver function and protein expression in the related pathways showed no significant effects (Figure 1D). Thus, ASX and olive oil had no significant effect on normal liver tissue.

**Figure 2:**
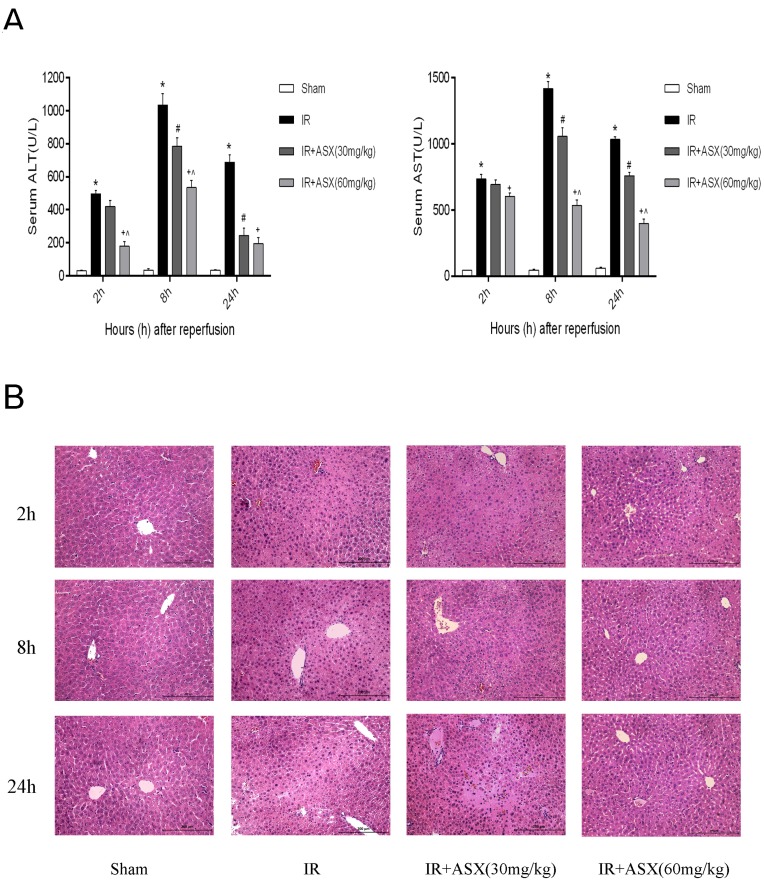
ASX pretreatment ameliorates hepatic IR injury. (**A**) ALT and AST were expressed as the mean ± SD of 6 mice per group. * *p* < 0.05 for IR *vs.* sham, ^#^ *p* < 0.05 for IR + ASX (30 mg/kg) *vs.* IR, ^+^ *p* < 0.05 for IR + ASX (60 mg/kg) *vs.* IR, ^ *p* < 0.05 for IR + ASX (60 mg/kg) *vs.* IR + ASX (30 mg/kg); (**B**) Representative hematoxylin and eosin (HE) stained liver sections are shown by digital microscopy. Original magnifications: 200×.

### 2.2. ASX Pretreatment Ameliorates Hepatic IR Injury Including Liver Enzymes and Pathology

ALT and AST are important indicators of liver dysfunction and are found in the hepatocyte cytoplasm. When necrosis occurs in liver cells, ALT and AST may rise rapidly and are sensitive measurements. In our experiment, 2 h, 8 h and 24 h were the main observation time points for measuring ALT, AST and pathological changes. The results showed that the enzymes in the IR group were significantly increased compared with the sham group at each point, and this was particularly evident at 8 h. Following ASX treatment (30 mg/kg or 60 mg/kg), ALT and AST decreased in a dose-dependent manner (Figure 2A). These findings suggested that ASX can protect liver function and this effect was dependent on dose. To illustrate this, we used HE staining to observe pathological changes in liver tissue sections. At 2 h, only a small amount of nuclear enrichment with no significant necrosis was observed in the IR group. However, disorganized liver form, complete destruction of cell structure, disordered lobular structure and marked hepatocyte necrosis were seen at 8 h and 24 h. The low ASX concentration (30 mg/kg) reduced the necrotic area, while the high ASX concentration (60 mg/kg) showed a greater protective effect (Figure 2B).

### 2.3. ASX Reduced the Release of Inflammatory Factors Including TNF-α and IL-6

**Figure 3:**
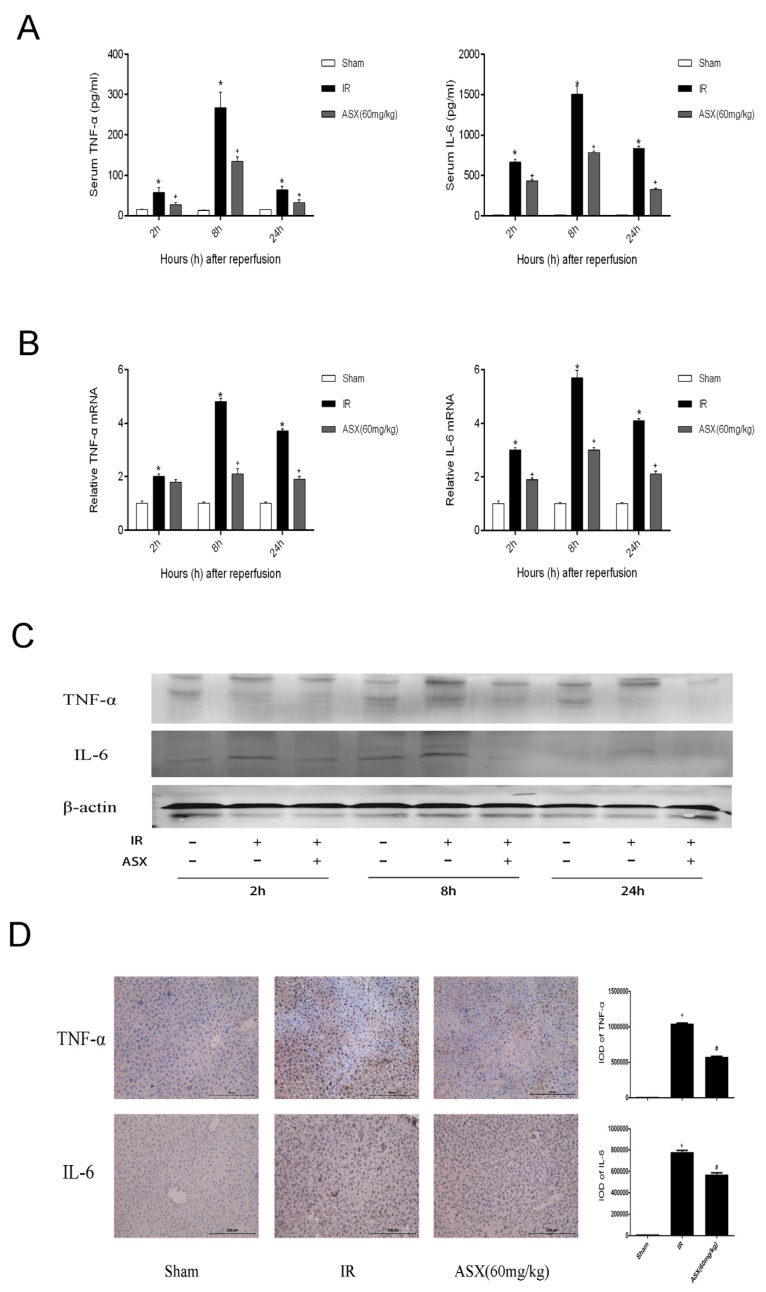
ASX reduced the release of TNF-α and IL-6. (**A**) The level plasma of TNF-α and IL-6 is shown as the mean ± SD of 6 mice per group. * *p* < 0.05 for IR *vs.* Sham, ^#^ *p* < 0.05 for ASX (60 mg/kg) *vs.* IR; (**B**) The mRNA expression of TNF-α and IL-6 was assessed by real-time PCR. The experiments were repeated three times and the data are shown as the mean ± SD. * *p* < 0.05 for IR *vs.* Sham, ^#^ *p* < 0.05 for ASX (60 mg/kg) *vs.* IR; (**C**) Western blots of the expression of TNF-α and IL-6 are shown by gray bands at each time point; (**D**) Representative immunohistochemistry staining shows the expression of TNF-α and IL-6 at 8 h. * *p* < 0.05 for IR *vs.* Sham, ^#^ *p* < 0.05 for ASX (60 mg/kg) *vs.* IR. Original magnifications: 200×.

Ischemia reperfusion of tissues can promote the release of oxygen free radicals and related inflammatory factors (TNF-α, IL-6) which aggravate the injury of endothelial cells leading to liver microcirculation disturbance via chemotaxis [2]. Therefore, we selected ASX at the dose of 60 mg/kg to determine TNF-α and IL-6 in the serum and tissues of mice, respectively, using ELISA, real time PCR, western blot and histochemical staining techniques. The results showed that the expression of TNF-α and IL-6 increased in the IR group not only in plasma but in the tissues. The ELISA results on serum levels demonstrated that TNF-α and IL-6 showed an increasing trend and peaked at 8 h. ASX reduced the release of TNF-α and IL-6 at each time point (Figure 3A). Figure 3B,C shows the gene and protein levels of TNF-α and IL-6. Compared with the sham group, the gene and protein levels of TNF-α and IL-6 increased in the IR group with the greatest effect of ASX seen at 8 h. We selected tissue slices at the 8 h period for immunohistochemical staining. The integral optical density of yellow granules was used to show severity, which was consistent with the mRNA and protein expression described above (Figure 3D). The results for plasma content, mRNA levels, protein expression and tissue staining provided strong evidence that ASX inhibited the production of inflammatory cytokines, such as TNF-α and IL-6, in serum and tissue.

### 2.4. ASX Alleviated Apoptosis and Autophagy by Reducing the Bax/Bcl-2 Ratio

**Figure 4:**
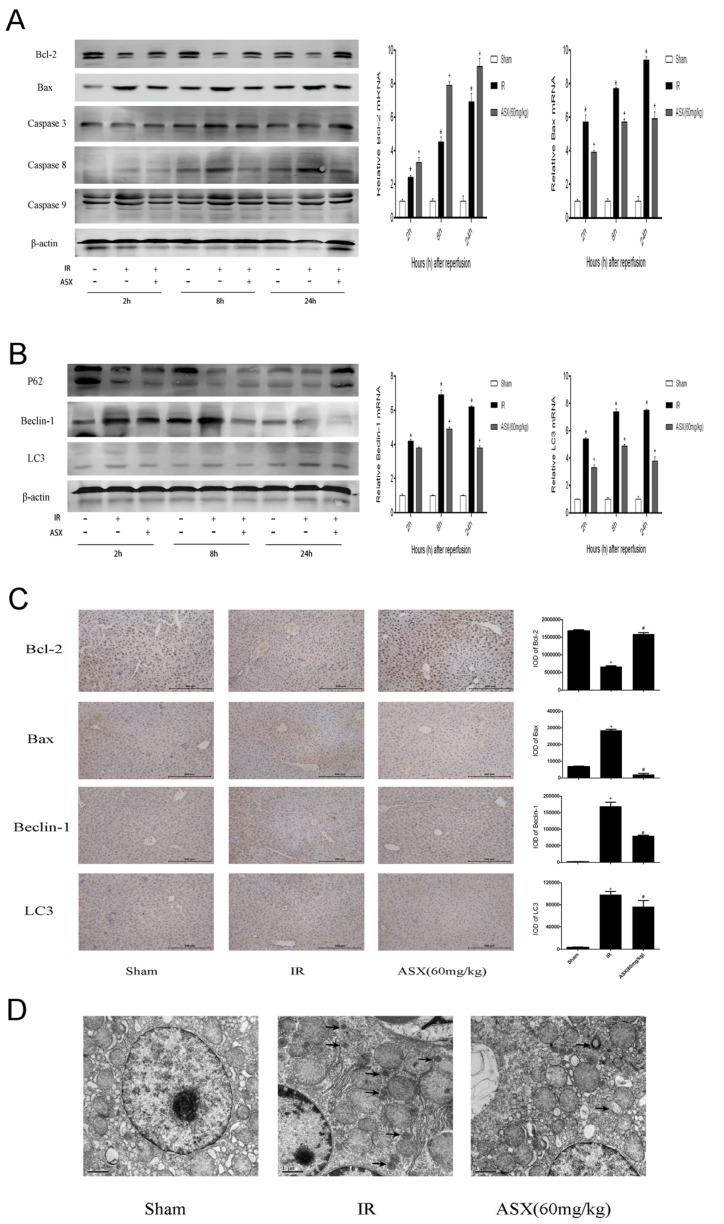
ASX reduced the proteins related to apoptosis and autophagy. (**A**) Protein expression of Bcl-2, Bax, Caspase-3, Caspase-8 and Caspase-9 was detected by western blots. The mRNA expression of Bcl-2 and Bax was detected by real-time PCR, and described as the mean ± SD. * *p* < 0.05 for IR *vs.* Sham, ^+^ *p* < 0.05 for ASX (60 mg/kg) *vs.* IR; (**B**) Western blots of P62, Beclin-1 and LC3 are shown by gray bands. The gene expression of Beclin-1 and LC3 were determined by real-time PCR. Data are described as the mean ± SD. * *p* < 0.05 for IR *vs.* Sham, ^+^ *p* < 0.05 for ASX (60 mg/kg) *vs.* IR; (**C**) Representative immunohistochemistry staining shows the expression of Bcl-2, Bax, Caspase-9, Beclin-1 and LC3 at 8 h. * *p* < 0.05 for IR *vs.* Sham, ^#^ *p* < 0.05 for ASX (60 mg/kg) *vs.* IR. Original magnifications: 200×; (**D**) Arrows indicate autophagosomes detected in liver tissue by transmission electron microscopy. Original magnifications: 10,000×.

Autophagy and apoptosis are preconditions which cause necrosis of the liver. Interrelation and distinction between them commonly play a key role in the ischemia reperfusion process. Therefore, we amplified the gene and assessed the protein expression of Bcl-2 and Bax during apoptosis, and Beclin-1 and LC3 during autophagy. ASX promoted the level of anti-apoptotic Bcl-2 but inhibited the pro-apoptotic Bax, leading to a decline in the ratio of Bax and Bcl-2 as shown by real-time PCR and western blot. Autophagy-related proteins, such as Beclin-1 and LC3, significantly increased due to disordered energy metabolism after IR and ASX reduced the damage caused by autophagy-induced cell necrosis (Figure 4A,B). These results were verified by histochemical staining (Figure 4C). In addition, detection of autophagosomes was carried out by electron microscopy (Figure 4D). Compared with the sham group, the autophagic vacuoles of the IR group were markedly increased. Following ASX administration, the agglutinated chromatin, damaged mitochondria and autophagy corpuscles were not easily seen. Taken together, these results demonstrated that ASX inhibited apoptosis and autophagy to protect hepatocytes from necrosis.

**Figure 5:**
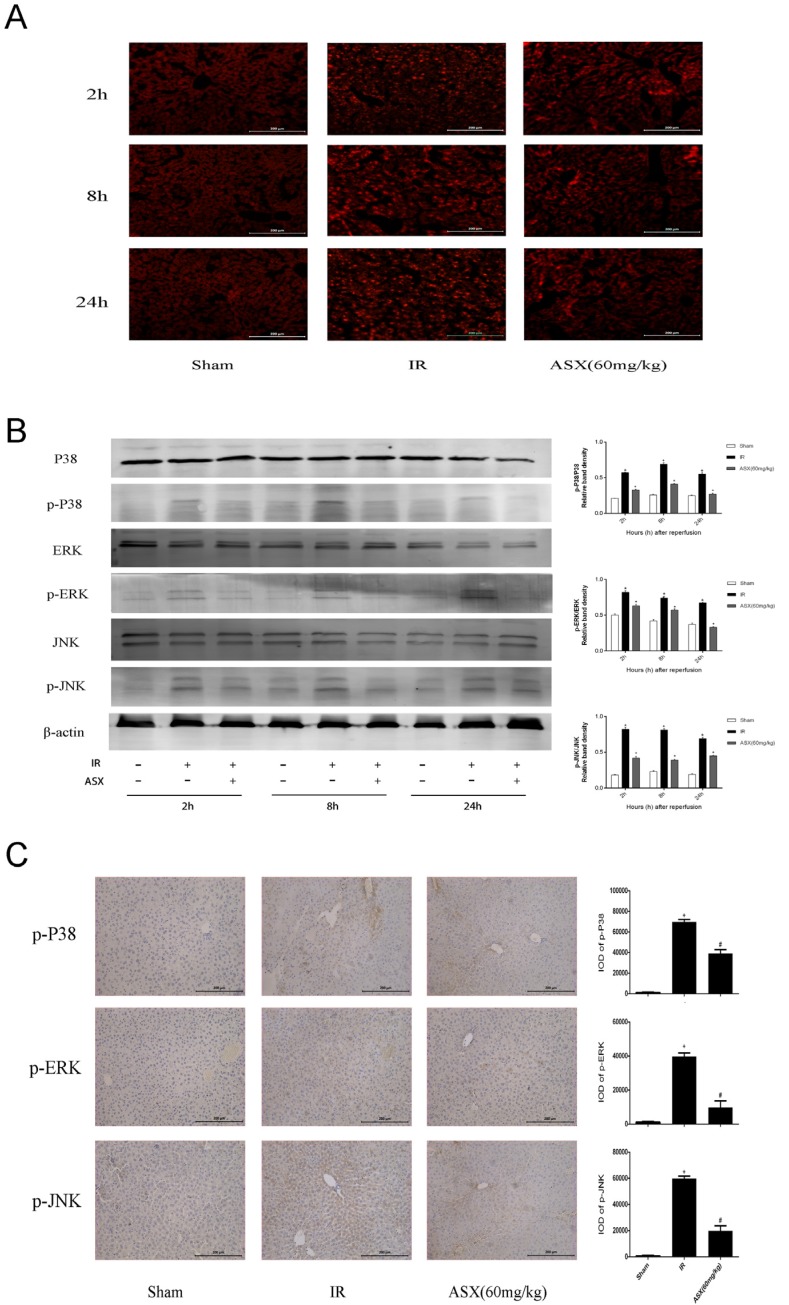
ASX inhibited ROS generation and the phosphorylation of proteins in the MAPK family. (**A**) The production of ROS was detected by the fluorescent probe, DHE. Bright red dot-like substances indicate strong ROS expression. Original magnification: 200×; (**B**) Protein expression of P38 MAPK, p-P38 MAPK, ERK, p-ERK, JNK, p-JNK was detected by western blots. The quantitative evaluation was determined by relative band density of phosphorylation levels. * *p* < 0.05 for IR *vs.* Sham, ^+^ *p* < 0.05 for ASX (60 mg/kg) *vs.* IR; (**C**) Representative immunohistochemistry staining shows the expression of p-P38 MAPK, p-ERK and p-JNK at 8 h. * *p* < 0.05 for IR *vs.* Sham, ^#^ *p* < 0.05 for ASX (60 mg/kg) *vs.* IR. Original magnification: 200×.

### 2.5. ASX Attenuates ROS/MAPK Signal Pathways by Inhibiting the Phosphorylation of P38 MAPK, ERK and JNK

Mitogen-activated protein kinase (MAPK) is expressed in all eukaryotic cells including P38 MAPK, ERK and JNK subgroups that control cell growth, differentiation, apoptosis and stress reactions to the environment [13]. The production of reactive oxygen species (ROS) after IR injury and the oxidative stress reaction are essential for apoptosis. We used the fluorescent probe, DHE, histochemical staining, and western blot to detect ROS and proteins related to the MAPK family. As shown in Figure 5A, ROS were dyed bright red by DHE compared with the sham group and showed strong expression in the IR group. After ASX treatment, reductions in bright red dot-like substances indicated less ROS production. In addition, P38 MAPK, ERK, JNK and their phosphorylation levels were measured to calculate the proportion of protein phosphorylation (Figure 5B). The results showed that the phosphorylation level significantly increased after IR, which was consistent with the expression of ROS. Following ASX administration, a downward trend in p-P38 MAPK, p-ERK and p-JNK expression in liver tissue was observed, which was consistent with immunohistochemical staining (Figure 5C). These results suggested that ASX inhibited ROS generation and the phosphorylation of key proteins related to the MAPK family, which may be an essential pathway in mitigating cell apoptosis and autophagy.

## 3. Discussion

The mechanism of hepatic IR has not been clarified due to its complexity, but increasing evidence shows that the production of ROS and inflammatory cytokines are key factors in inducing liver damage [40]. ASX, a natural antioxidant from marine organisms, may improve the outcome of liver transplantation.

We demonstrated that ASX and its solvent had no negative effects on liver function and pathology in normal liver tissue. Thus, the effect of ASX on ROS and cytokines induced by hepatic IR injury requires further investigation. The sharp rise in ALT and AST is an important manifestation of our successful IR model. Serum levels of liver enzymes declined significantly, which was consistent with structural disorders, and HE staining showed the presence of necrosis. We selected an ASX dose of 60 mg/kg to assess TNF-α and IL-6 which mediate cell apoptosis and necrosis at the gene and protein levels. ASX inhibited both TNF-α and IL-6, which has been described by previous researchers. Kupffer cells, known as hepatic macrophages, are activated by IR to produce ROS, TNF-α, IL-6 and other highly reactive molecules that stimulate T and B cells and mediate the adhesion of white blood cells, platelets and liver sinusoidal endothelial cells, causing aggravation of the hepatic microcirculation [41,42]. In addition, the activation of NF-κB by TNF-α also increased inflammation leading to injury [43]. These effects resulted in ALT and AST being released from ruptured cell cytoplasm, and their plasma levels corresponded with the extent of cellular damage [44]. ASX can scavenge radicals at the outer and inner parts of the cell membrane, due to its unique molecular structure with hydroxyl and keto moieties on each ionone ring [45,46]. ASX provides protection by reducing oxygen free radicals and pro-inflammatory factors. However, the pathways which play a role in ASX hepatocyte protection by avoiding oxidative stress have not yet been clarified.

Mitogen-activated protein kinase (MAPK), originally purified from fat cells by Ray and Sturgill in 1988, has been described as four distinct subgroups: P38 MAPK, extracellular signal-regulated kinase (ERK), c-jun *N*-terminal kinase (JNK) and big MAPK 1 (BMK1) [47,48]. The first three subgroups are usually activated concurrently in animals. In resting cells, the MAPK family, distributed in the cytoplasm, is activated by phosphorylation or inactivated by MAPK phosphatases (MKPs), a class of dual-specificity phosphatases (DSPs) as negative regulators in MAPK pathway, in order to migrate to the nucleus and cell membrane, resulting in the regulation of gene transcription [49,50]. P38 MAPK is an important component of the MAPK family which can be activated by extracellular stress, including oxidative stress, pro-inflammatory cytokines, ultraviolet radiation, and heat shock [14,51,52,53]. P38 activation also stimulates monocyte-macrophages to produce TNF-α, IL-6 as well as increased nitric oxide (NO) accelerated ROS. Inhibition of P38 has a significant protective effect on multiple systems. Kim *et al.* showed that ASX inhibited apoptotic cell death in neural progenitor cells via modulation of the P38 and MEK signaling pathways [51]. Systemic inhibition of P38 MAPK weakened ongoing pulmonary inflammation, as described by Nick and colleagues [53]. Another effective advantage of MAPK inhibition has been shown in type II diabetes, Crohn’s disease, acute colitis and other systems [52,54,55]. In hepatic IR injury, P38 MAPK in addition to ERK and JNK participates in the injury induced by ROS and cytokines. Koike and Hashimoto reduced IR injury following heart, lung and liver transplantations by adding P38 MAPK inhibitors [56,57]. In our study, we also conducted comprehensive examinations of mRNA and protein levels. The results showed that activated phosphorylated P38 MAPK (p-P38 MAPK) increased due to ROS and cytokines caused by IR, and its phosphorylation reduced after ASX treatment. This shows that ASX inhibited the P38 MAPK pathway by reducing ROS and TNF-α. Activation levels of ERK and JNK have also been consistently validated in previous studies [13,14,58,59,60]. We consider that ASX weakened phosphorylation of the MAPK family and provided protection by scavenging ROS and inactivating Kupffer cells which release inflammatory factors.

**Figure 6:**
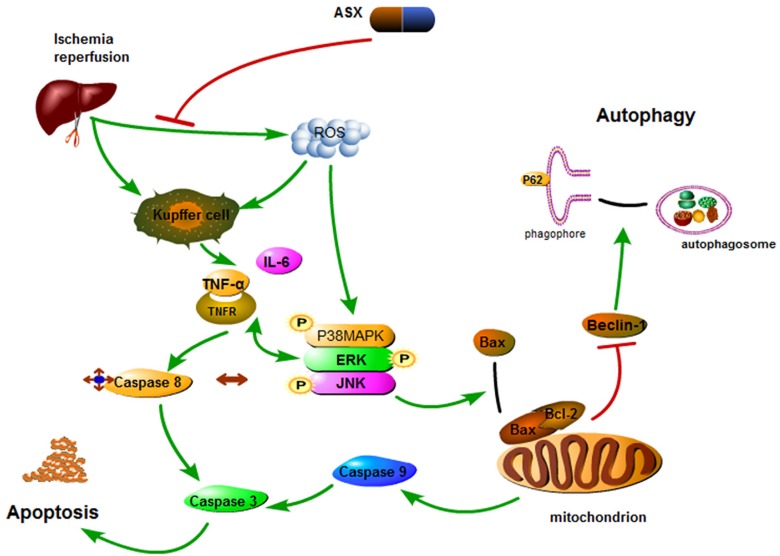
Mechanism of action of astaxanthin. In hepatic IR, astaxanthin weakened phosphorylation of the MAPK family and provided protection by scavenging ROS and inactivating Kupffer cells which release inflammatory factors. MAPK pathways activated by TNF-α and IL-6 can not only activate capase-8 but also phosphorylate Bcl-2 to induce caspase-9 activation. Inactive Bcl-2 released Beclin-1 that enhanced autophagy. Thus, astaxanthin inhibits the release of ROS and cytokines during hepatic IR to offer protection by inhibiting apoptosis and autophagy.

How does inhibition of P38 MAPK protect the liver from damage? Studies have found that P38 not only enhanced cleaved caspase-8 mediated apoptosis induced by TNF-α, but also had an effect on the Bcl-2 family resulting in increased intrinsic apoptosis [47]. The release of ROS and cytokines hindered mitochondrial energy metabolism, and stimulated related enzymes that could phosphorylate P38 MAPK. Phosphorylated P38 MAPK transferred Bax located in the cytoplasm of normal hepatocytes to mitochondria, forming dimers that had a direct influence on the membrane permeability transition pore (MPTP) [61,62]. Open MPTPs triggered mitochondrial permeability transition (MPT) to release cytochrome C and then activate caspase-9 resulting in apoptosis. ERK and JNK pathways activated by TNF-α and IL-6 can phosphorylate Bcl-2 (inactive form) to prevent inhibition of cell apoptosis [63,64]. These three pathways eventually interact with each other to activate cleaved caspase-3, leading to liver cell apoptosis [65]. The effect of ASX on the elimination of ROS and TNF-α was evaluated by PCR, western blot and immunohistochemistry staining. The key proteins in the MAPK family, such as P38, ERK, JNK, Bcl-2, Bax, and Caspases, were determined and their expression levels assessed. In addition, autophagy was found to be involved in hepatic IR injury. A reduction in related oxidative stress factors decreased the activity of Bax in mitochondrial transfer, which mediated free active Bcl-2. Bcl-2 containing Bcl-2 homology 3 (BH3)-only domains can be combined with Beclin-1 to inhibit autophagy [66]. Inhibition of autophagy limited the conversion of LC3-I to LC3-II and the formation of autophagosomes and P62, and the autophagy-related transporter combined with mature LC3 via its special ubiquitin-binding domains was detected, as shown in Figure 6 [67]. Thus, we observed increased Beclin-1 and LC3 and reduced P62 in the ASX-treated group.

In summary, we preliminarily found that activation of the MAPK family mediated by ROS and cytokines which induced apoptosis and autophagy could be inhibited by ASX during hepatic IR injury in mice. The link between related drugs and pathways requires further investigation in order to identify safer and effective therapies for hepatic IR injury due to its complex mechanism.

## 4. Experimental Section

### 4.1. Animals

All experimental protocols carried out on mice were approved by the Animal Care and Use Committee at Shanghai Tongji University. Male Balb/C mice weighing 20–25 g (6–8 weeks old) were housed in a clean room at a constant temperature (22–25 °C) and a 12 h:12 h light-dark cycle. The mice were purchased from Shanghai SLAC Laboratory Animal Co. Ltd. (Shanghai, China) and given food and water *ad libitum*. The study was performed in accordance with the standards established by the recommendations of the Guide for the National Science Council of the Republic of China.

### 4.2. Preparation of Reagents

ASX was purchased from Sigma-Aldrich (St. Louis, MO, USA) and stored in the dark at −20 °C. The antibodies used in this study included those against Bcl-2, Bax, Caspase-3, Caspase-8, Caspase-9, LM3, TNF-α, IL-6, p-JNK (all from Cell Signaling Technology, Danvers, MA, USA), P62, Beclin1, JNK, P38 MAPK, p-P38 MAPK, β-actin (all purchased from Proteintech, Chicago, IL, USA), ERK, and p-ERK (both from Epitomics, Burlingame, CA, USA). ROS Fluorescent Probe-DHE was purchased from Vigorous (Beijing, China). TNF-α and IL-6 Enzyme Linked Immunosorbent Assay (ELISA) kits were acquired from eBioscience (San Diego, CA, USA).

### 4.3. Experimental Design

The mice were randomly distributed to one of six groups as follows:

Group I, control group (*n* = 8): mice received saline by gavage

Group II, oil group (*n* = 8): mice received olive oil by gavage

Group III, ASX group (*n* = 16): mice received ASX (30 mg/kg or 60 mg/kg) by gavage

Group IV, sham group (*n* = 18): mice received olive oil by gavage without IR

Group V, IR group (*n* = 18): mice received olive oil by gavage before IR

Group VI, IR + ASX group (*n* = 36): mice received ASX (30 mg/kg or 60 mg/kg) by gavage before IR

ASX was dissolved in olive oil to obtain a suspension and stored in the dark at 4 °C. The mice were given saline, olive oil or ASX for 14 consecutive days. All mice in Group I, II, III and IV were killed after 14 days, while, six mice were randomly selected from Group IV, V and VI (6 mice in each dose) were sacrificed at 2 h, 8 h or 24 h after IR. Serum and liver tissues were obtained for further experiments.

### 4.4. Mouse Model of Hepatic Ischemia-Reperfusion Injury

A mouse model of 70% hepatic warm ischemia was established according to a previously reported method [68]. Mice were fasted for 24 h before surgery, but allowed water. The mice were anesthetized by an intraperitoneal standard dosage (1.25%) of sodium pentobarbital (Nembutal, St. Louis, MO, USA). The abdominal viscera were identified after entering the intra-abdominal cavity along the medioventral line. The middle and left hepatic lobes, and then, the first porta hepatis were dissociated from adjacent structures using wet cotton swabs. Hepatic ischemia was achieved by occluding the hepatic artery, portal vein and bile duct using vascular clamps to obtain 70% tissue ischemia. When the hepatic lobe showed a color change from crimson to light red, the incision was covered with humid saline gauze for 60 min. The abdominal incision was then sewn without vascular clamps. To maintain a constant body temperature, we applied an animal body temperature maintenance instrument (ZS Dichuang, Beijing, China).

### 4.5. ALT and AST Enzyme-Activity and Cytokine Measurements

Sera were isolated by centrifuging at 4500× *g* for 10 min after storing for 4–5 h at 4 °C. Fifty microliters aliquots of serum were placed in Eppendorf (EP) tubes and stored at −80 °C. Serum ALT and AST were measured by an automated chemical analyzer (Olympus AU1000, Tokyo, Japan). The plasma levels of TNF-α and IL-6 were assessed using ELISA kits, according to the manufacturer’s protocol.

### 4.6. Histopathological Evaluation

The removed liver tissue was fixed with 4% paraformaldehyde for at least 24 h, and then dehydrated using different concentrations of ethanol. After immersion in xylene, the samples were waxed in paraffin blocks to prevent crystallization. Sections 5 μm thick were cut and stored at room temperature. The sections were stained with hematoxylin-eosin (HE) for observation under a light microscope.

### 4.7. Western Blot Analysis

Fresh liver tissues were cut into pieces and immediately stored in liquid nitrogen. Tissue blocks of approximately 100 mg were disintegrated by radioimmunoprecipitation assay (RIPA) lysis buffer containing protease inhibitors (PI) and phenylmethane-sulfonyl fluoride (PMSF). The extracted protein was mixed with loading buffer after bicinchoninic acid (BCA) quantification and stored at −20 °C. According to the standard curve, the same amount of protein was added to sodium dodecyl sulfate (SDS)-polyacrylamide gels (8%–12.5%) for gel electrophoresis, and then transferred onto 0.22 μm polyvinylidene fluoride (PVDF) membranes. Bovine serum albumin (BSA, 5%) was used to close all nonspecific sites of the membranes at least 1 h before dilute primary antibodies (Bcl-2, 1:500; Bax, 1:500; Caspase-3, 1:500; Caspase-8, 1:1000; Caspase-9, 1:500; IL-6, 1:500; TNF-α, 1:500; LC3, 1:1000; Beclin-1, 1:500; JNK, 1:1000; p-JNK, 1:500; p-ERK, 1:1000; ERK, 1:500; p-P38, 1:500; P-38, 1:1000 and β-actin, 1:1000) were combined with the proteins at 4 °C. The next day, phosphate buffer solution containing 0.1% Tween 20 (PBST) was used to wash the membranes three times before and after incubation with the secondary antibodies (horseradish peroxidase-conjugated anti-rabbit or anti-mouse IgG) in the proportion of 1:2000 for 1 h at 37 °C. The strength of the protein signals was detected using the Odyssey two-color infrared laser imaging system (LI-COR Biosciences, Lincoln, NE, USA).

### 4.8. Immunohistochemical Staining

The slices were dried in a drying oven for 2 h and then immersed in dimethyl benzene for dewaxing. Different concentrations of ethanol were used to dehydrate the prepared sections. After washing with phosphate buffer solution (PBS) three times, antigen retrieval was then performed with citrate buffer by heating to 95 °C for 10 min and cooling to room temperature (four cycles). In order to inhibit endogenous peroxidase activity, we added 3% hydrogen peroxide to the sections at room temperature for 20 min and then blocked them with 5% BSA solution. The liver slices were then incubated with antibodies directed against Bcl-2 (1:100), Bax (1:500), Beclin-1 (1:100), LC3 (1:500), p-JNK (1:50), p-ERK (1:100), p-P38 MAPK (1:50) for 24 h at 4 °C and with a secondary antibody (1:50) for 1 h at 37 °C after washing. A diaminobenzidine (DAB) kit was used to show granular brown substances which were observed and captured by a microscope with a digital camera (Leica Wetzlar, Germany). Image Pro Plus Software 6.0 (Media Cybernetics, Silver Spring, MD, USA) was used to calculate the integrated optical densities (IOD) of the sections.

### 4.9. SYBR Green Real-Time Polymerase Chain Reaction (PCR)

Approximately 100 mg of tissue were removed from liquid nitrogen and triturated in mortars soaked with diethyl pyrocarbonate (DEPC) treated water. Trizol, chloroform and isopropyl alcohol were added to the tissues for extraction of total RNA. After determination of the purity and concentration, a reverse transcription kit (TaKaRa Biotechnology, Dalian, China) was used to transcribe RNA into cDNA. We used a 10 μL reaction volume containing primers and related enzymes for SYBR Green quantitative real-time PCR using a 7900HT fast real-time PCR system (Applied Biosystems, NewYork, NY, USA), according to the Premix EX Taq protocols (TaKaRa Biotechnology, Dalian, China). Gene expression was calculated on the basis of the solubility curve and the ratios of target genes. All primers used in the experiments are shown in Table 1.

**Table 1:** Nucleotide sequences of primers used for qRT-PCR.

Gene		Primer Sequence (5′–3′)
*TNF-α*	Forward	CAGGCGGTGCCTATGTCTC
	Reverse	CGATCACCCCGAAGTTCAGTAG
*IL-6*	Forward	CTGCAAGAGACTTCCATCCAG
	Reverse	AGTGGTATAGACAGGTCTGTTGG
*LC3*	Forward	GACCGCTGTAAGGAGGTGC
	Reverse	AGAAGCCGAAGGTTTCTTGGG
*Beclin1*	Forward	ATGGAGGGGTCTAAGGCGTC
	Reverse	TGGGCTGTGGTAAGTAATGGA
*Bax*	Forward	AGACAGGGGCCTTTTTGCTAC
	Reverse	AATTCGCCGGAGACACTCG
*Bcl-2*	Forward	GCTACCGTCGTCGTGACTTCGC
	Reverse	CCCCACCGAACTCAAAGAAGG
*β-actin*	Forward	GGCTGTATTCCCCTCCATCG
	Reverse	CCAGTTGGTAACAATGCCATGT

### 4.10. Transmission Electron Microscopy

Liver tissues were prefixed by immersion in 3% glutaraldehyde and 0.2% mol/L sodium cacodylate for 4 h and fixed in 1% osmium tetroxide for 1 h. Autophagosomes were observed by transmission electron microscopy (LEO 906E, Oberkochen, Germany).

### 4.11. Reactive Oxygen Species (ROS) of Liver Tissue Assay

The ROS fluorescent probe, dihydroethidium (DHE), can penetrate the cell membrane and is oxidized by ROS to produce a red fluorescence. Fresh liver tissues were fixed with 4% formaldehyde and then dehydrated using 30% sucrose solution overnight at 4 °C. The tissues were embedded using opti-mum cutting temperature compound (OCT) and cut into sections 5 μm thick. After adequate washing, the sections were incubated with DHE (10 μM) for 60 min in the dark. The sections were then washed with PBS three times, for 10 min each time. Red light stimulated by green light was acquired by the fluorescence microscope for calculation of the red area.

### 4.12. Statistical Analysis

All data are presented as the mean ± standard deviation (SD) and analyzed using SPSS 20.0 software (Chicago, IL, USA). Differences among groups were detected by one-way analysis of variance (ANOVA) using the Student-Newman-Keuls (SNK) method. *p* < 0.05 was considered statistically significant.

## 5. Conclusions

Pretreatment with astaxanthin attenuated hepatic ischemia reperfusion-induced apoptosis and autophagy via the ROS/MAPK pathway in mice, and the relationship between the two may be associated with the reduction of inflammatory cytokines.
